# Supplementary material for: RNA editing of Filamin A pre‐mRNA regulates vascular contraction and diastolic blood pressure
Source: EMBO J. 2018 Aug 7;37(19):e94813. doi: 10.15252/embj.201694813 (PMC6166124; doi:10.15252/embj.201694813)
Supplement: Supplementary file 1 — Appendix [file EMBJ-37-e94813-s001.pdf]

Table of contents:

Figures and Tables	Page no.
Figure S1	1
Figure S2	2
Figure S3	3
Figure S4	4
Figure S5	6
Figure S6	7
Figure S7	8
Figure S8	9
Figure S9	10
Table S1a	14
Table S1b	16
Table S2	18

Figure S1

A.

B.

C.

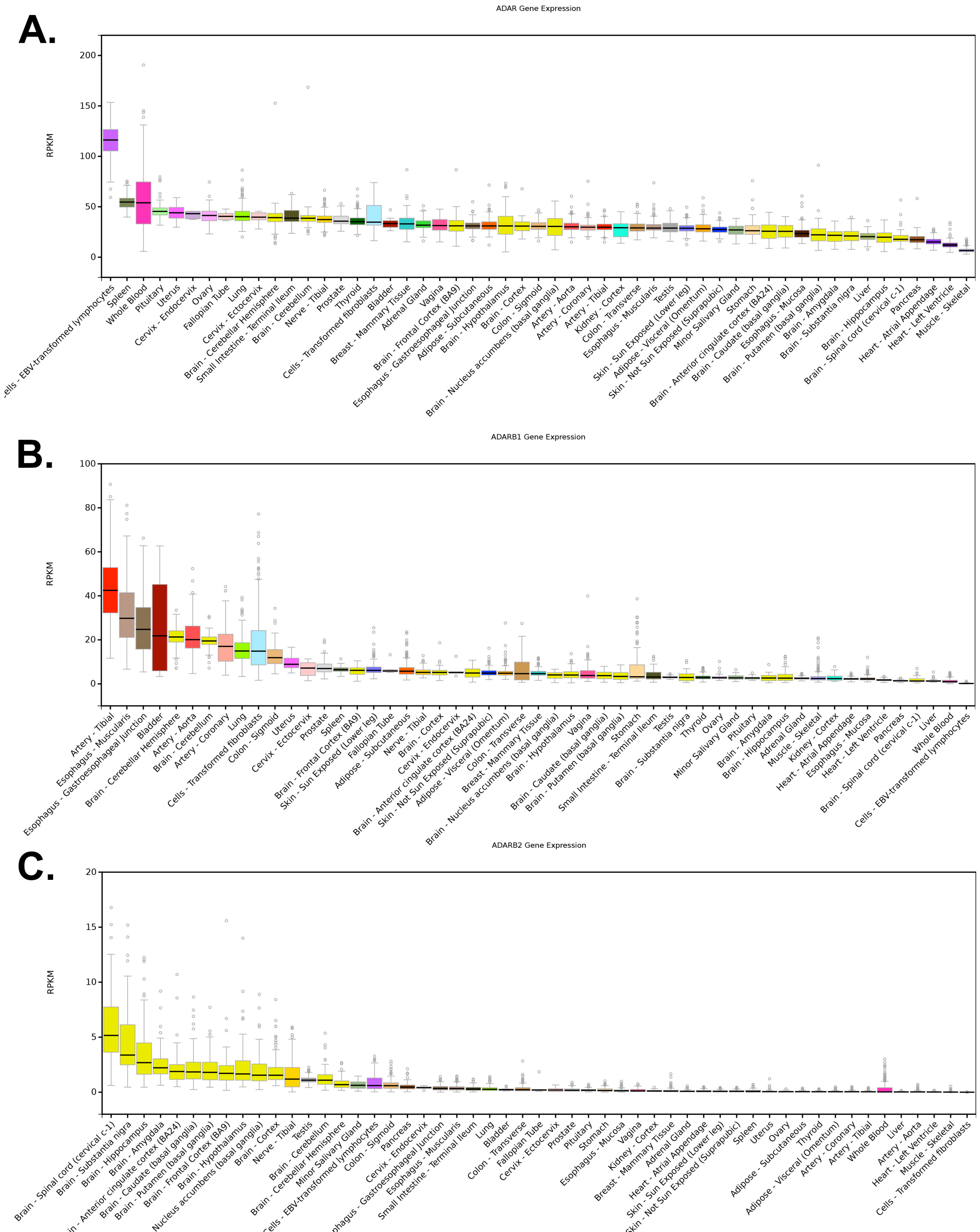

ADAR1 and ADAR3 expression across tissues

(A) **ADAR1 expression is homogeneous across tissues**. Unlike ADAR2 expression, Adar1 expression is evenly distributed across tissues and in general has higher expression levels than ADAR2. No large differences are observed between several human tissues analyzed.

(B) **ADAR2 is most strongly expressed in vascular tissues**. Graph showing ADAR2 gene expression data derived from GTEx for all the represented human tissues. Note the highest expression of ADAR2 in the tibial artery.

(C) **ADAR3 expression is brain specific**. Expression of ADAR3 is lower than ADAR1 and ADAR2 expression and is limited to the brain. All figures were filtered as described for ADAR2 expression. Data was adapted from GTEx portal.

Figure S2

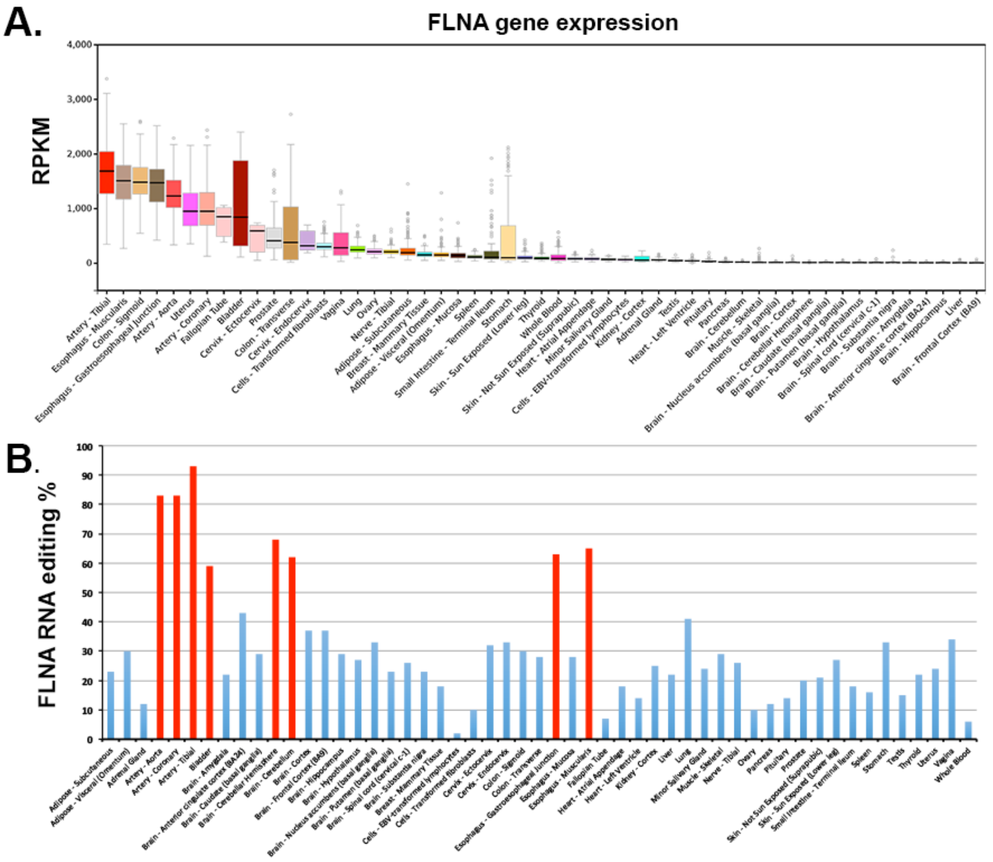

(**A**) **FLNA gene expression** from over 50 different tissues in a total of >500 different donors using GTEx data. Boxes represent the 25th and the 75th percentile with median represented by the black line in the box. The whiskers depict the minimum and the maximum value.

(B) Bar graph shows **FLNA RNA editing** (%) among several human tissues. Note very high editing levels in the arterial system.

Figure S3

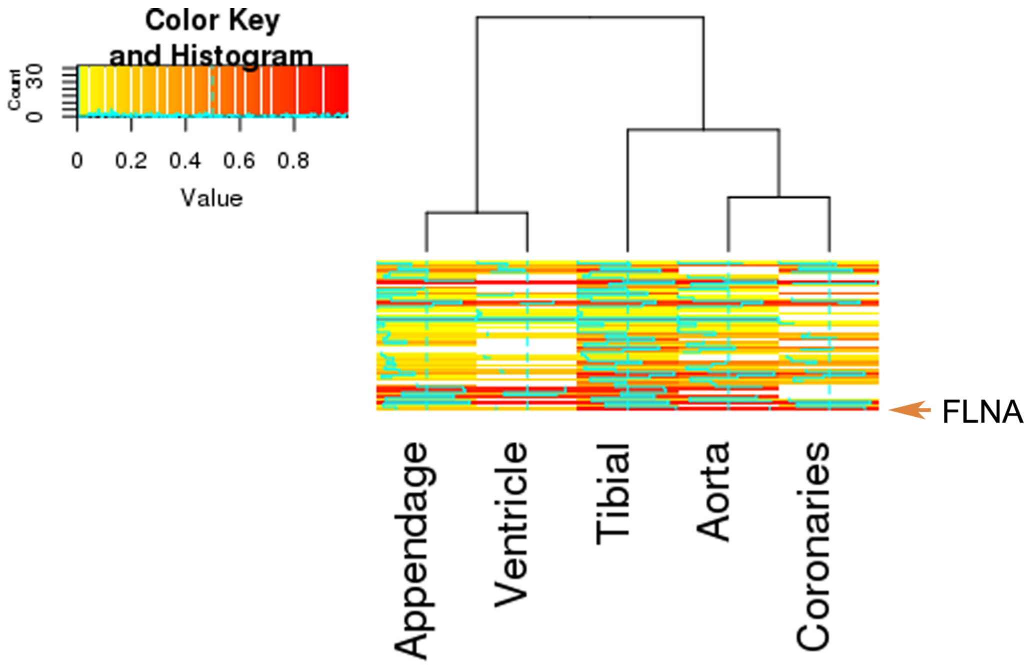

Abundant editing in the cardiovascular tissue. Cluster analysis of editing sites and editing levels shows clustering of the ventricle, appendage as well as the tibial artery, dorsal aorta, and coronaries. Editing levels are indicated as yellow (low) to red (high) and low read coverage is indicated in turquoise. Note that FLNA is highly expressed throughout showing medium editing levels in the ventricle and very high editing levels in the tibial and dorsal aorta and coronaries. FLNA is the bottom most line marked by arrowhead.

Figure S4

A.

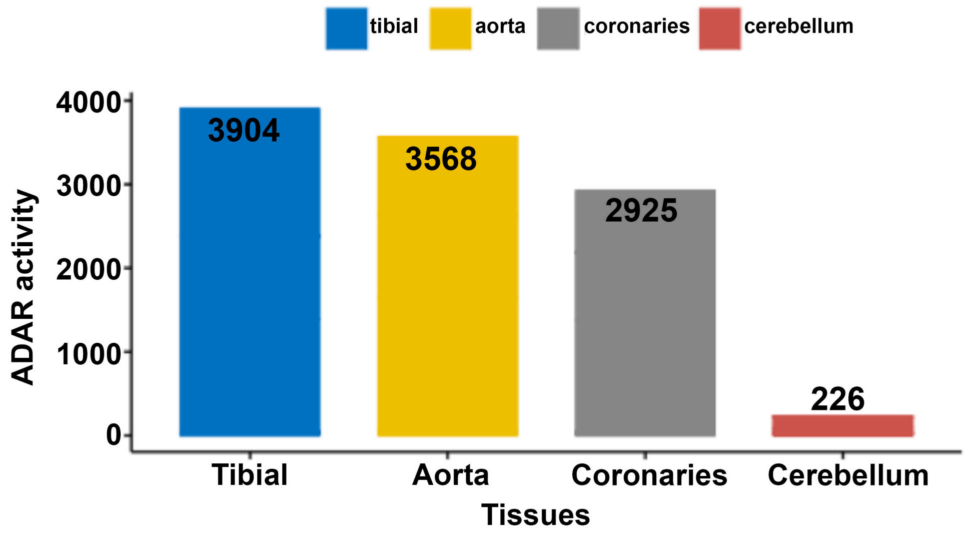

B.

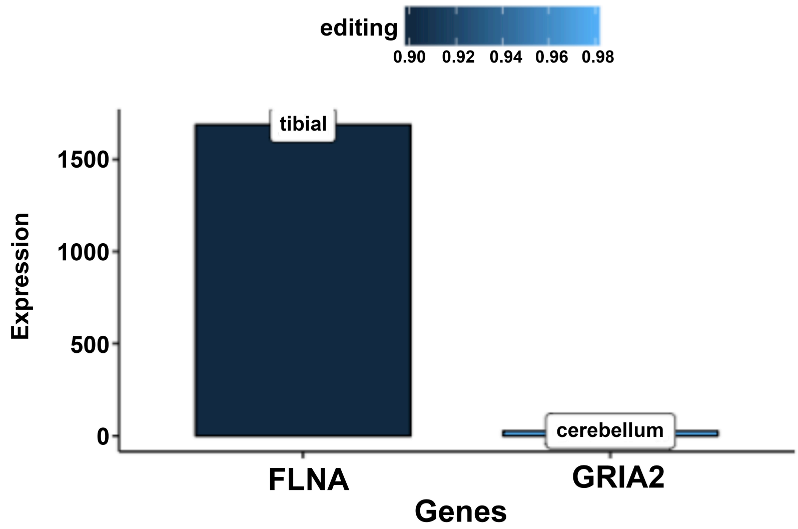

C.

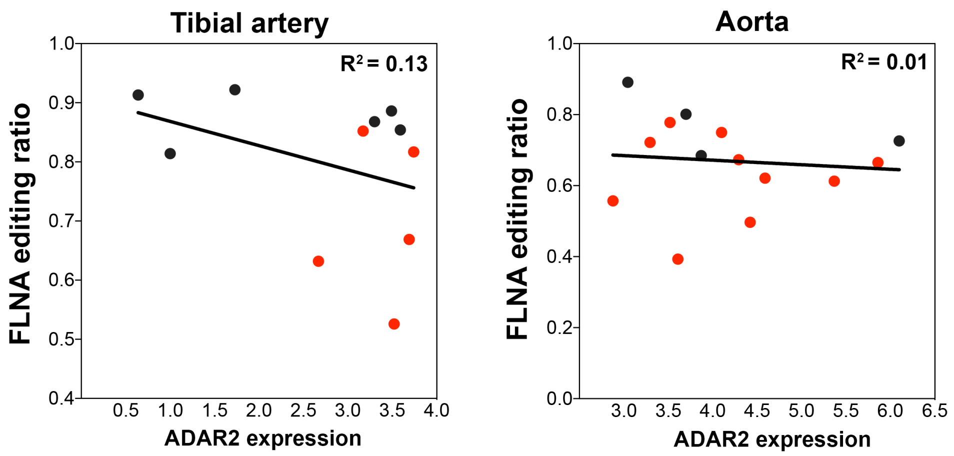

ADAR activity in cardiovascular tissues is higher than in other tissues, including brain

(A) Comparison of ADAR activity in vascular tissues and brain. Total ADAR editing activity data was calculated by determining median substrate expression levels from GTEx and median editing levels from REDIportal for 38 conserved prominent editing sites in the tissues investigated (Picardi, D'Erchia et al., 2017). As seen, the editing in arteries exceeds editing activity in the brain by a full order of magnitude.

(B) Total editing of FLNA in tibial artery versus GRIA2 editing in the cerebellum. The glutamate receptor in the cerebellum is considered a prominently edited site. Graph shows that FLNA in the tibial artery is edited to a similar extent (color filling of the bars) but much more expressed than the glutamate receptor (GRIA2) in the cerebellar hemisphere. This means that the actual editing activity invested in FLNA in the arterial system far exceeds that which takes place in what is considered the hallmark site of editing so far- the AMPA glutamate receptor in the brain.

(C) Correlation plots of ADAR2 expression and FLNA editing levels in human tibial artery and aorta samples from healthy and diseased individuals. Y-axes depicts relative editing levels in FLNA mRNA. X-axis depicts relative ADAR2 expression. Black dots represent control samples and red dots represent diseased patient samples**.** No correlation was observed between FLNA editing levels and ADAR2 expression. At least 4 control and 7 diseased human donors were used in each case.

Figure S5

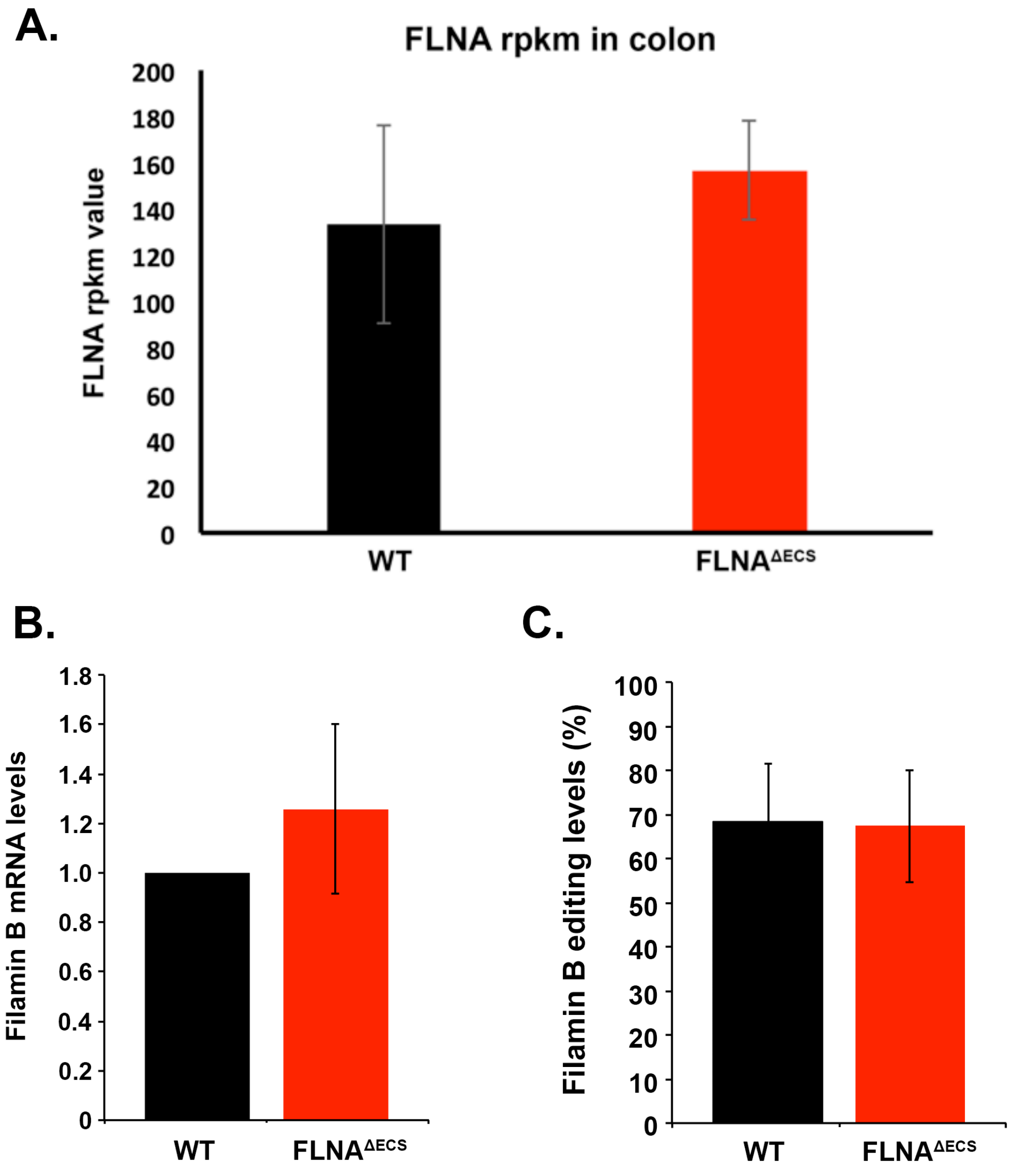

(A) RPKM values of FLNA do not change in wt and FLNA$^{\Delta ECS}$ mice. RNA sequencing was performed on 3 wt and 3 FLNA$^{\Delta ECS}$ mice samples of distal colon where FLNA editing is high. RPKM values were calculated for all samples and average and standard deviation of RPKM values are plotted for wt and FLNA$^{\Delta ECS}$ mice. **(B,C) Expression levels and editing levels of FLNB pre-mRNA are not altered in the absence of FLNA editing (B)** qRT-PCR was performed on RNA extracted from aortic arch of wild-type and FLNA$^{\Delta ECS}$ mice and compared to tubulin mRNA. Expression levels of FLNB showed no significant change. **(C)** Similarly, editing levels in FLNB remained unaffected in the absence of FLNA pre-mRNA editing. Data represent mean ± SD from three independent experiments. $P<0.05$ was considered significant.

Figure S6

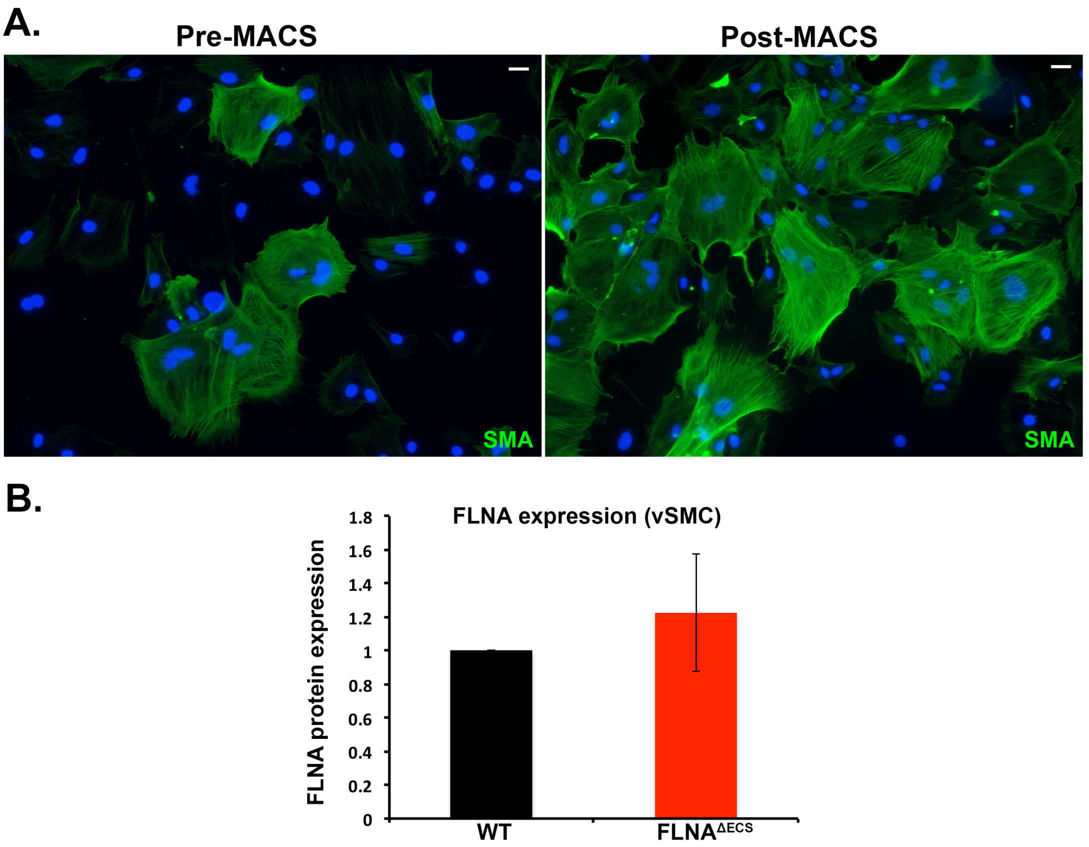

(A) Depletion of fibroblasts by MACS purification enriches for vascular smooth muscle cells. Images showing SMA (Smooth muscle actin) staining (green) of primary vascular smooth muscle cells pre- and post magnetic depletion of fibroblasts. Nuclei (blue) were marked by DAPI. Scale bar: 20 μm. **(B)** Graph showing FLNA protein expression determined by western blotting in primary vascular smooth muscle cells (vSMC). Y axis represent FLNA protein expression normalized to tubulin levels. Data shown as mean ± SD from three independent experiments. The differences are non significant with P>0.05.

Figure S7

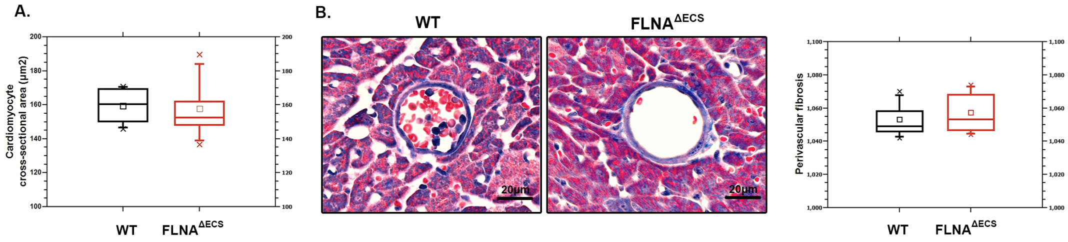

21 day old FlnA$^{\Delta ECS}$ mice have normal cardiac development and cardiomyocyte diameter.

(A) Box plot showing cardiomyocyte area measurements. (B) Representative heart sections stained with Masson trichrome staining and box plot showing quantification of collagen deposition (bright blue) indicating that young hearts containing unedited FLNA do not develop perivascular fibrosis. Five male mice were analyzed for each genotype. Scale bar: 20µm.

Figure S8

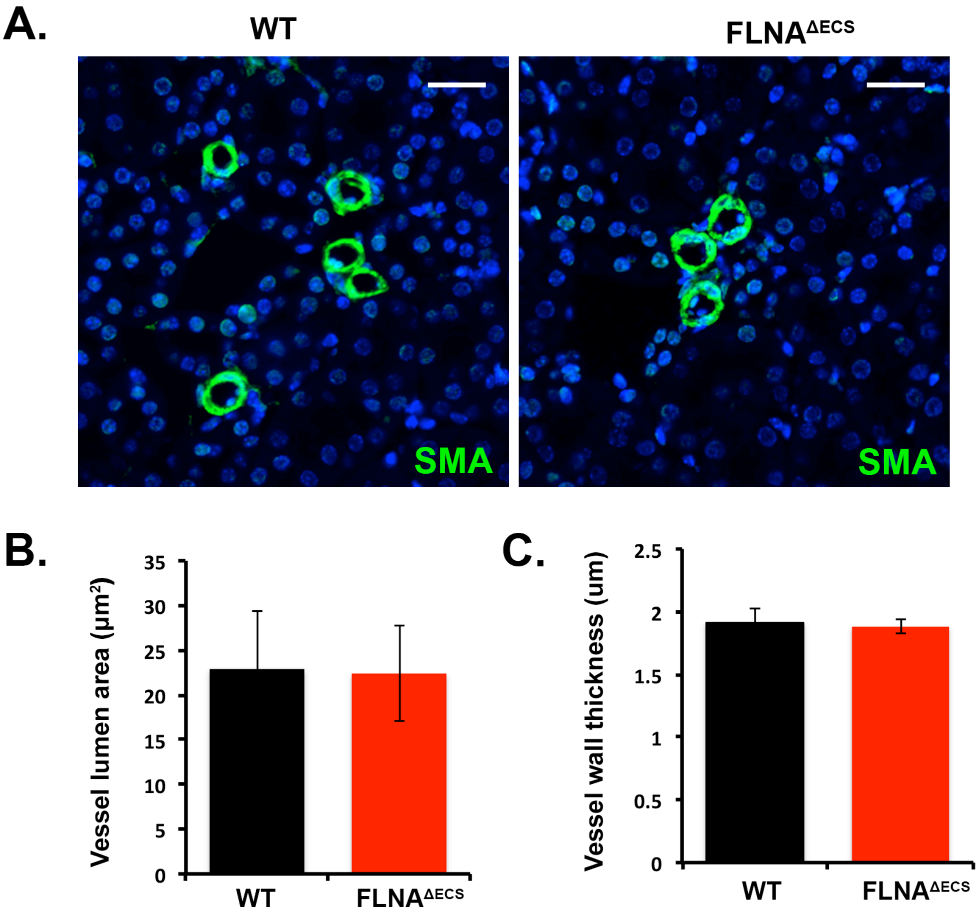

Microvasculature remodeling in FLNA$^{\Delta ECS}$ mice

(A) Kidney sections showing SMA staining in green, nuclei are marked in blue. Scale bar = 30μm. Graphs showing **(B)** vessel lumen area and **(C)** vessel wall thickness in wt and FLNA$^{\Delta ECS}$ mice. None of the parameters showed significant difference. 5 wt and 4 FLNA$^{\Delta ECS}$ mice were used for analysis. $P<0.05$ was considered significant.

Figure S9

A.

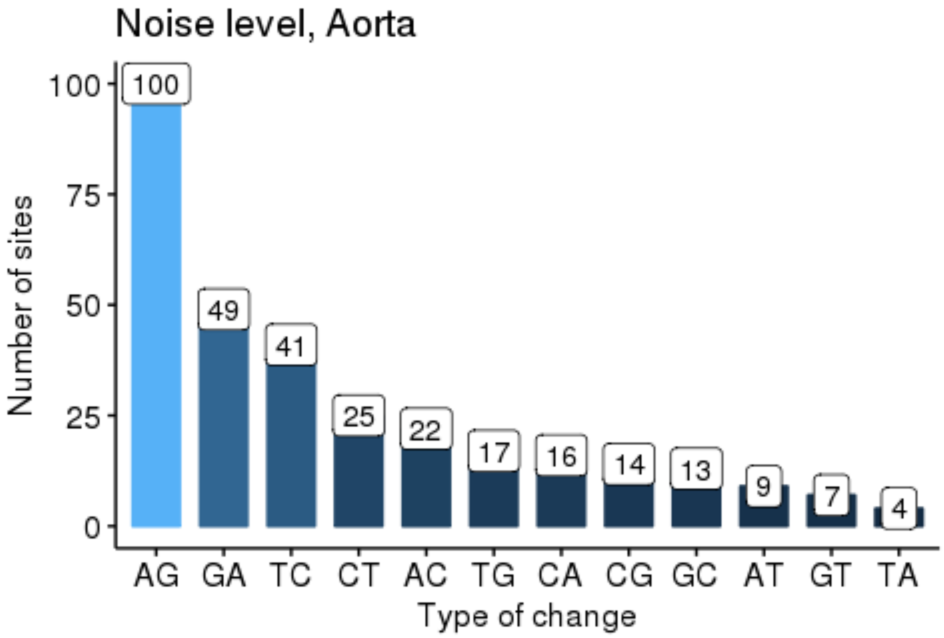

Noise level, Aorta
Number of sites
100
75
50
25
0
100
49
41
25
22
17
16
14
13
9
7
4
AG GA TC CT AC TG CA CG GC AT GT TA
Type of change

B.

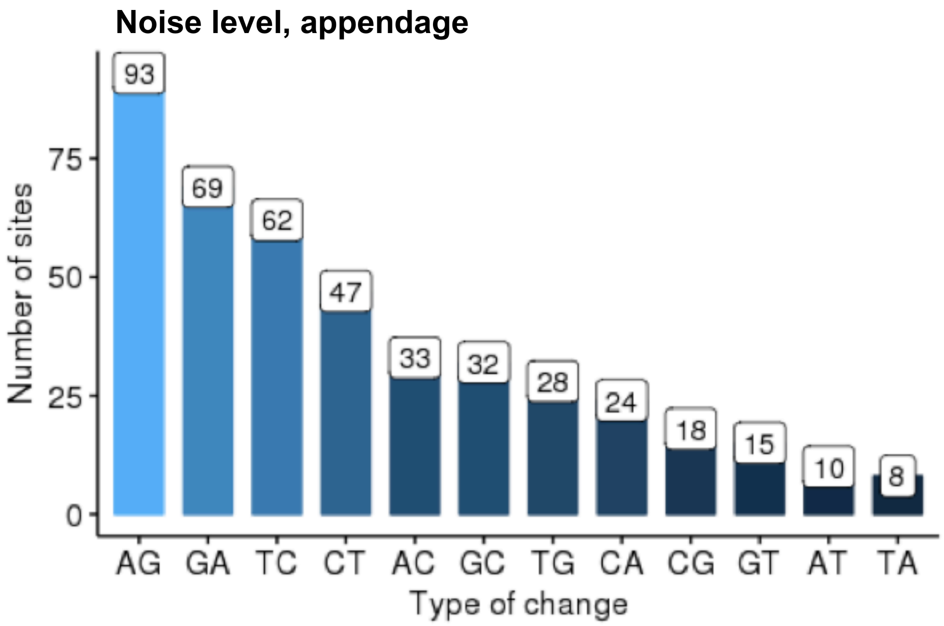

Noise level, appendage
Number of sites
75
50
25
0
93
69
62
47
33
32
28
24
18
15
10
8
AG GA TC CT AC GC TG CA CG GT AT TA
Type of change

C.

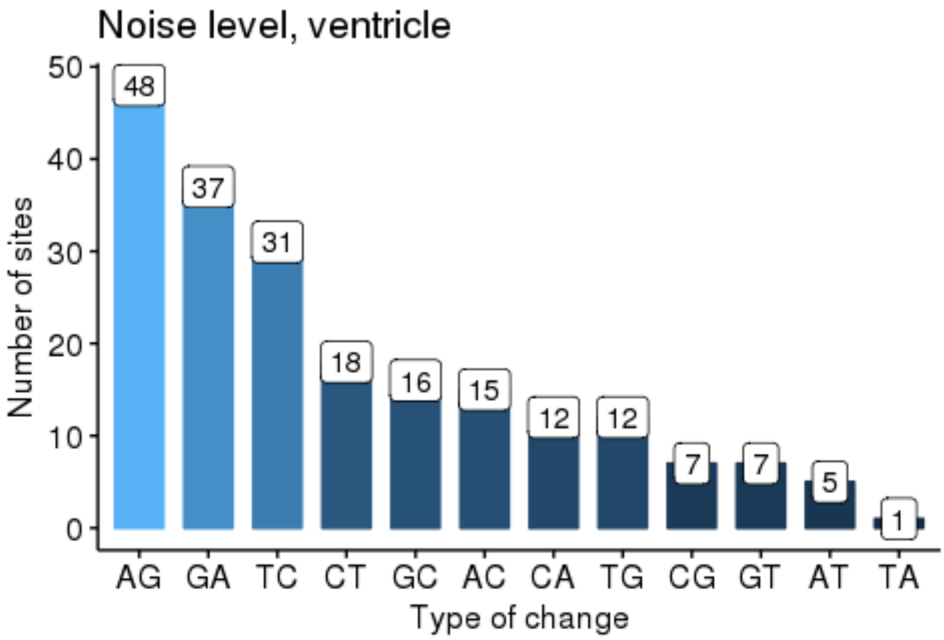
Noise level, ventricle
Number of sites
0
10
20
30
40
50
48
37
31
18
16
15
12
12
7
7
5
1
AG GA TC CT GC AC CA TG CG GT AT TA
Type of change

D.

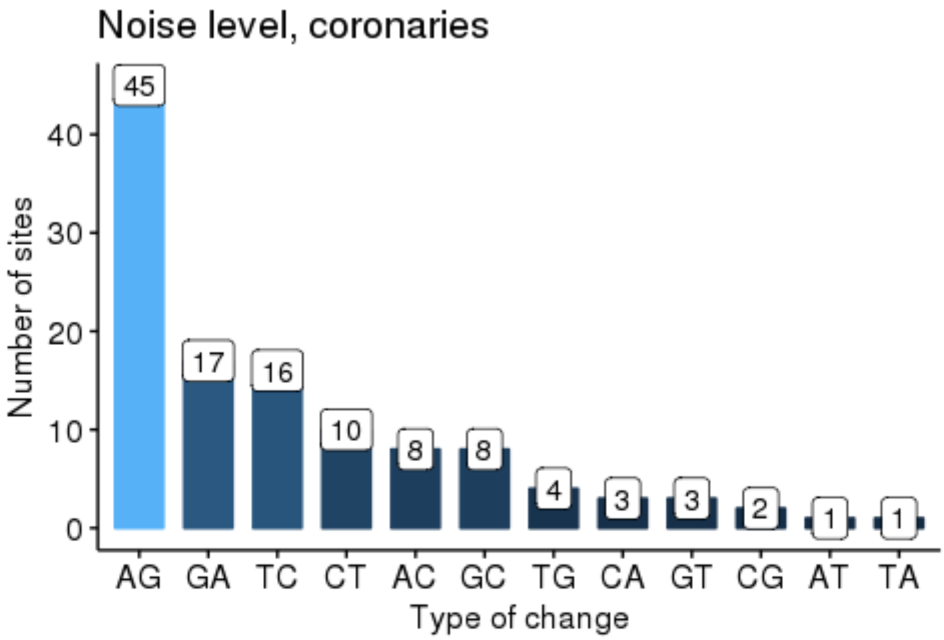
Noise level, coronaries
Number of sites
0
10
20
30
40
45
17
16
10
8
8
4
3
3
2
1
1
AG GA TC CT AC GC TG CA GT CG AT TA
Type of change

E.

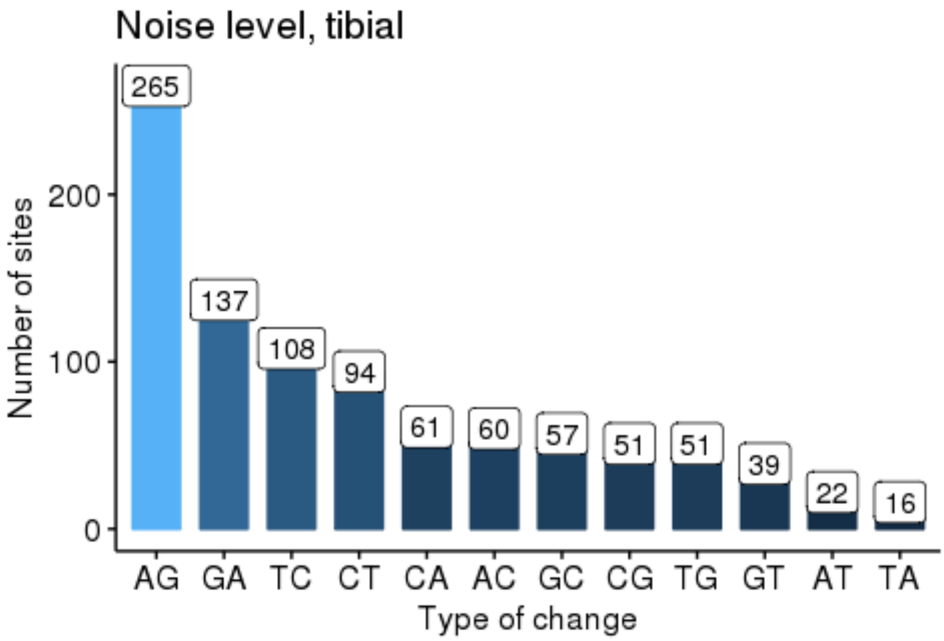

Estimation of noise per tissue. Represented are all types of nucleotide exchanges at the selected sites (before filtering out highly variable and therefore less credible sites such as immune genes). The level of noise is calculated by the ratio of the second most common change to the most common change (A to G). The tissue type is depicted at the top of each graph.

References:

Picardi E, D'Erchia AM, Lo Giudice C, Pesole G (2017) REDIportal: a comprehensive database of A-to-I RNA editing events in humans. Nucleic Acids Res 45: D750-D757

Table S1a: The top 15 ADAR editing sites in cardiovascular tissue and cerebellum

Postion 57976234 in the IGFBP7 gene is most heavily edited in all vascular tissue and is most likely the most edited site in the human body.
The second most edited site is located at position 153579950 in the actin crosslinking FLNA gene.
FLNA is most heavily edited in the tibial artery.
Editing of Gria2 is much less abundant.

	chr	pos	gene	tissue	Expression (	Editing level	adar.activity
1	chr4	57976234	IGFBP7	aorta	1830.11	0.87	15,92,196
**2**	**chrX**	**153579950**	**FLNA**	**tibial**	**1687.66**	**0.9**	**15,18,894**
3	chr4	57976234	IGFBP7	tibial	1414.94	0.95	13,44,193
4	chr4	57976234	IGFBP7	coronaries	1408.9	0.95	13,38,455
5	chr4	57976286	IGFBP7	tibial	1414.94	0.69	97,63,086
6	chr4	57976286	IGFBP7	aorta	1830.11	0.53	96,99,583
7	chrX	153579950	FLNA	aorta	1231.87	0.76	93,62,212
8	chr4	57976286	IGFBP7	coronaries	1408.9	0.57	8,03,073
9	chrX	153579950	FLNA	coronaries	951.65	0.77	73,27,705
10	chr5	156736808	CYFIP2	cerebelum	49.05	0.69	3,38,445
11	chr6	102372589	GRIK2	cerebelum	35.19	0.85	2,99,115
12	chr6	102337702	GRIK2	cerebelum	35.19	0.76	2,67,444
13	chr4	158257875	GRIA2	cerebelum	26.3	0.98	25,774
14	chr1	160302244	COPA	aorta	42.28	0.59	2,49,452
15	chr6	34100903	GRM4	cerebelum	149.25	0.14	20,895

Table S1b: Editing of FLNA and IGFBP7 exceeds all other editing events by an order of magnitude.
When editing of the most edited sites is considered across several tissues, FLNA and IGFBP7 editing levels exceed editing levels at all other sites by an order of magnitude. Editing of the relatively abundant Gria 2 editing site ranks at position 9.

	Site	Gene	Total.activity
1	chr4_57976234	IGFBP7	42,74,844
2	chrX_153579950	FLNA	31,87,886
3	chr4_57976286	IGFBP7	2749.34
4	chr1_160302244	COPA	6,38,092
5	chr5_156736808	CYFIP2	5,56,973
6	chr4_158257875	GRIA2	31,364
7	chr6_102372589	GRIK2	3,00,061
8	chr6_102337702	GRIK2	2,68,324
9	chr4_158281294	GRIA2	2,35,913
10	chr21_34923319	SON	2,23,647

Table S2: changes in editing levels in healthy individual and dilated cardiomyopathy patients

Relative editing levels are given for dilated cardiomyopathy samples (dcm) and healthy samples (healthy mean AG)

P value of the change in editing is given

Number of samples for each condition are indicated (num_healtyh, num_diseased).

From this, the false discovery rate is calculated (FDR)

	chr	pos	dcm_mean.AG.	healthy_mean.AG.	editing.pval	num_healthy	num_disea	fdr	gene	func
1	chr1	160302244	0.178830357142857	0.312253521126761	1.51E-05	71	112	6.05E-06	COPA	nonsynonymous SNV
2	chr21	34923319	0.0612818181818182	0.155296296296296	2.50E-06	54	110	5.00E-07	SON	synonymous SNV
3	chrX	153579950	0.120705357142857	0.231347826086957	2.24E-08	69	112	2.51E-09	FLNA	nonsynonymous SNV
4	chr15	75646086	0.666785714285714	0.757	2.51E-08	70	112	2.51E-09	NEIL1	nonsynonymous SNV
5	chr19	46841490	0.0874579439252336	0.124138461538462	0.000286281208506138	65	107	0.00229024966804911	HIF3A	nonsynonymous SNV
6	chr15	75646087	0.368486486486486	0.439441176470588	0.00879437212734638	68	111	0.0586291475156426	NEIL1	synonymous SNV
7	chr19	46841538	0.0984414414414414	0.108246153846154	0.123057278132497	65	111	0.70318444647141	HIF3A	nonsynonymous SNV
8	chr3	58141791	0.211738738738739	0.247458333333333	0.275762454250763	72	111	1	FLNB	nonsynonymous SNV
9	chr11	65636053	0.984576576576577	0.977534246575342	0.280848151372035	73	111	1	EFEMP2	nonsynonymous SNV
10	chr1	22186113	8.26E+09	0	0.292624431351093	61	109	1	HSPG2	nonsynonymous SNV
11	chr19	5587279	0.000125	0	0.464154270094717	62	112	1	SAFB2	nonsynonymous SNV
12	chr1	46751578	0.000153153153153153	0	0.473556074829435	59	111	1	LRRC41	synonymous SNV
13	chr9	37429814	0.920919642857143	0.905945205479452	0.494876989956531	73	112	1	GRHPR	synonymous SNV
14	chr4	57976234	0.260205357142857	0.281246575342466	0.782004410427028	73	112	1	IGFBP7	nonsynonymous SNV
15	chr6	29911916	0.169135135135135	0.169267605633803	0.991944617542252	71	111	1	HLA-A	nonsynonymous SNV
