# Supplementary material for: RNA editing of Filamin A pre‐mRNA regulates vascular contraction and diastolic blood pressure
Source: EMBO J. 2018 Aug 7;37(19):e94813. doi: 10.15252/embj.201694813 (PMC6166124; doi:10.15252/embj.201694813)
Supplement: Supplementary file 2 — Expanded View Figures PDF [file EMBJ-37-e94813-s002.pdf]

Expanded View Figures

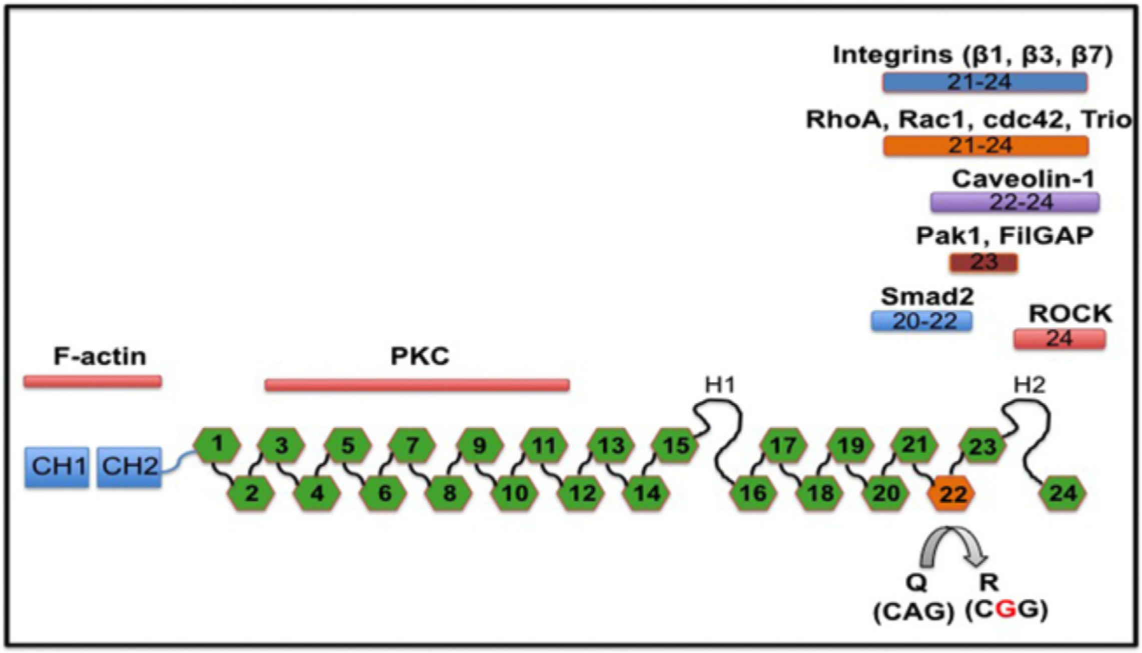

Figure EV1. Scheme of Filamin A structure and editing-induced amino acid exchange.

Filamin A protein is composed of 24 Ig-like repeats. Editing changes Q2341R amino acid in repeat 22 from a glutamine (Q) to an arginine (R), as indicated. Interaction partners to the respective regions are indicated above. The amino terminus harbors an actin-binding domain, while the C-terminal end is interacting with integrins, RhoA, Caveolin, Pak1, Smad2, or ROCK. Repeat 24 is required for homo- and heterodimerization with Filamin B.

 © 2018 The Authors

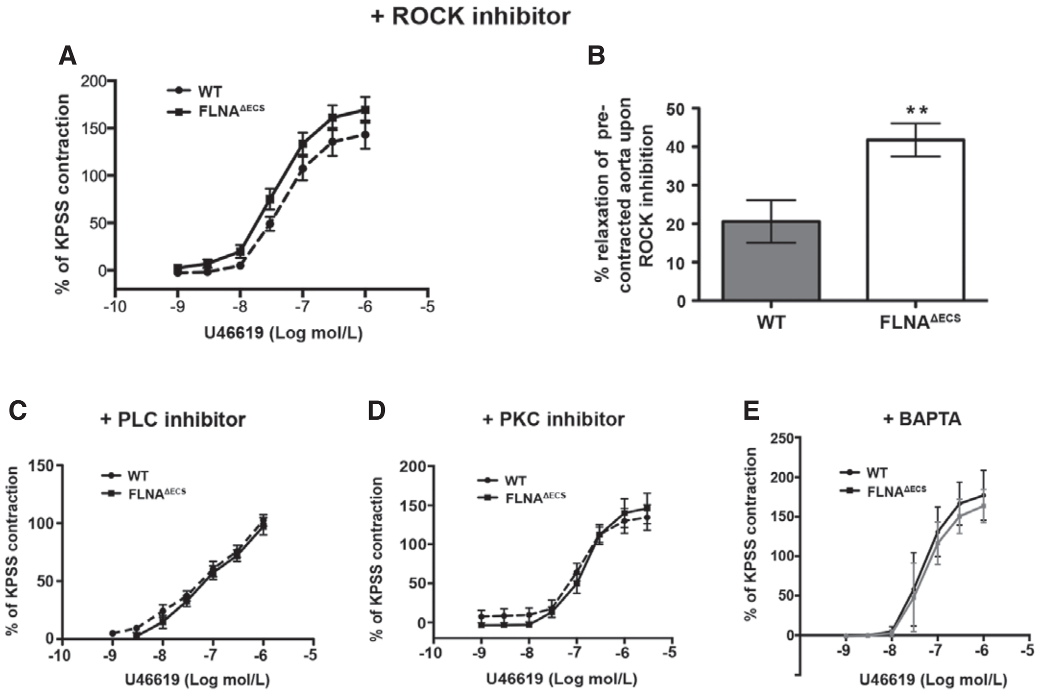

Figure EV2. Inhibition with either ROCK or PLC and PKC inhibitor abrogates the hypercontraction phenotype in FLNA$^{\Delta ECS}$ aortae.

- A Pre-inhibition with ROCK inhibitor before U46619 treatment diminishes the increased contraction in FLNA$^{\Delta ECS}$ aortae.
- B Treatment with the ROCK inhibitor following U46619 pre-contraction leads to increased relaxation in FLNA$^{\Delta ECS}$ aortae. $^{**}P < 0.01$ (Student's *t*-test).
- C–E Pre-inhibition with PLC inhibitor (C), PKC inhibitor (D), BAPTA (E) before U46619 treatment shows similar effects as ROCK inhibitor (A) abrogating the genotype-specific difference.

Data information: For each condition, 8–12 aortic rings from at least four wild-type (wt) and 4 FLNA$^{\Delta ECS}$ mice were used. Data are shown as mean ± SEM.
Source data are available online for this figure.

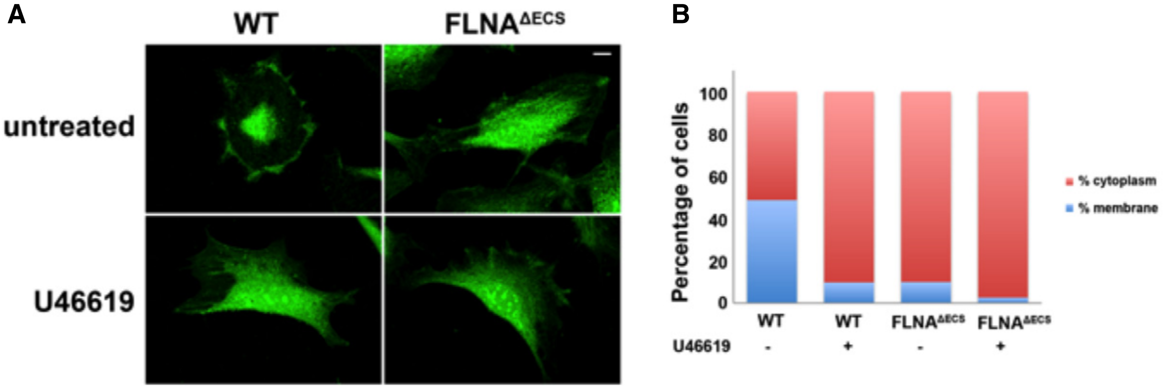

Figure EV3. FLNA editing-deficient vascular smooth muscle cells show mislocalization of p190^{RhoGAP}.

- A Representative images of wild-type (wt) and FLNA$^{\Delta ECS}$ vascular smooth muscle cells (vSMCs) stained with P190^{RhoGAP} antibody (green) before and after treatment with 1 μM U46619. Note the change in localization in wt cells after U46619 treatment that looks similar to the untreated cells expressing only unedited FLNA. Scale bar: 20 μm.
- B Quantification of subcellular localization of p190^{RhoGAP} in wt and FLNA$^{\Delta ECS}$ vSMCs. Graphs are plotted as percentage of cells showing membranous vs. cytoplasmic localization. Data were pooled after counting 250–300 cells in each condition.

Source data are available online for this figure.

© 2018 The Authors

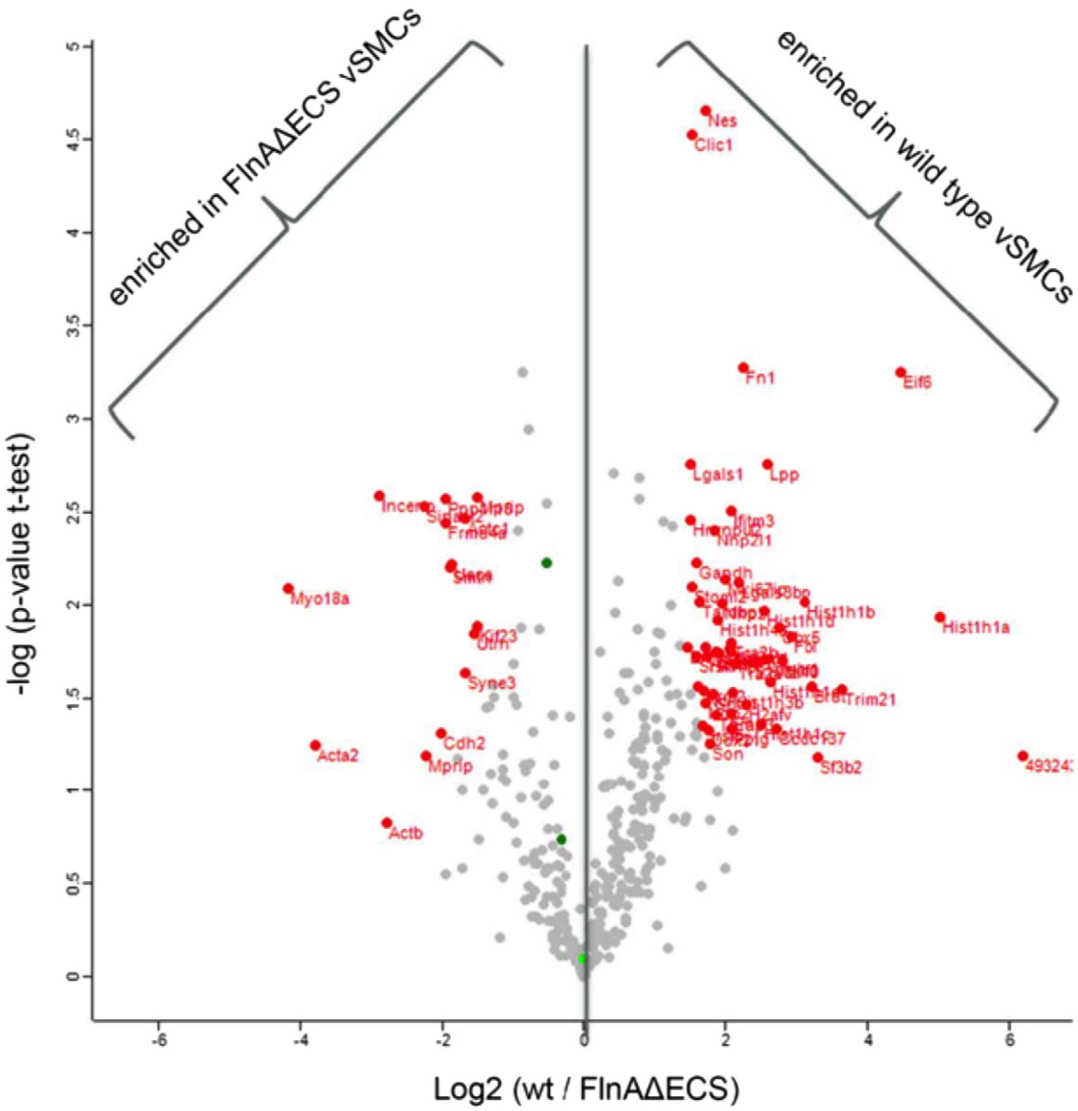

Figure EV4. Volcano plot.

Volcano plot presenting proteins differentially enriched in either of the two sets of Filamin A pull-down from $FLNA^{\Delta ECS}$ and wild-type vSMCs. *X*-axis represents mean log2 ratio of protein intensities between the two sets, plotted against the negative logarithm of the *P*-value from triplicate *t*-tests. Proteins marked in red on the left side of central line were enriched in $FLNA^{\Delta ECS}$ vSMCs pull-downs while the ones on the right side were enriched in wild-type vSMC pull-downs ($P < 0.05$; fold change > 2; modified Student's *t*-test). Total counts were first normalized to Filamin A in each data set.

 © 2018 The Authors

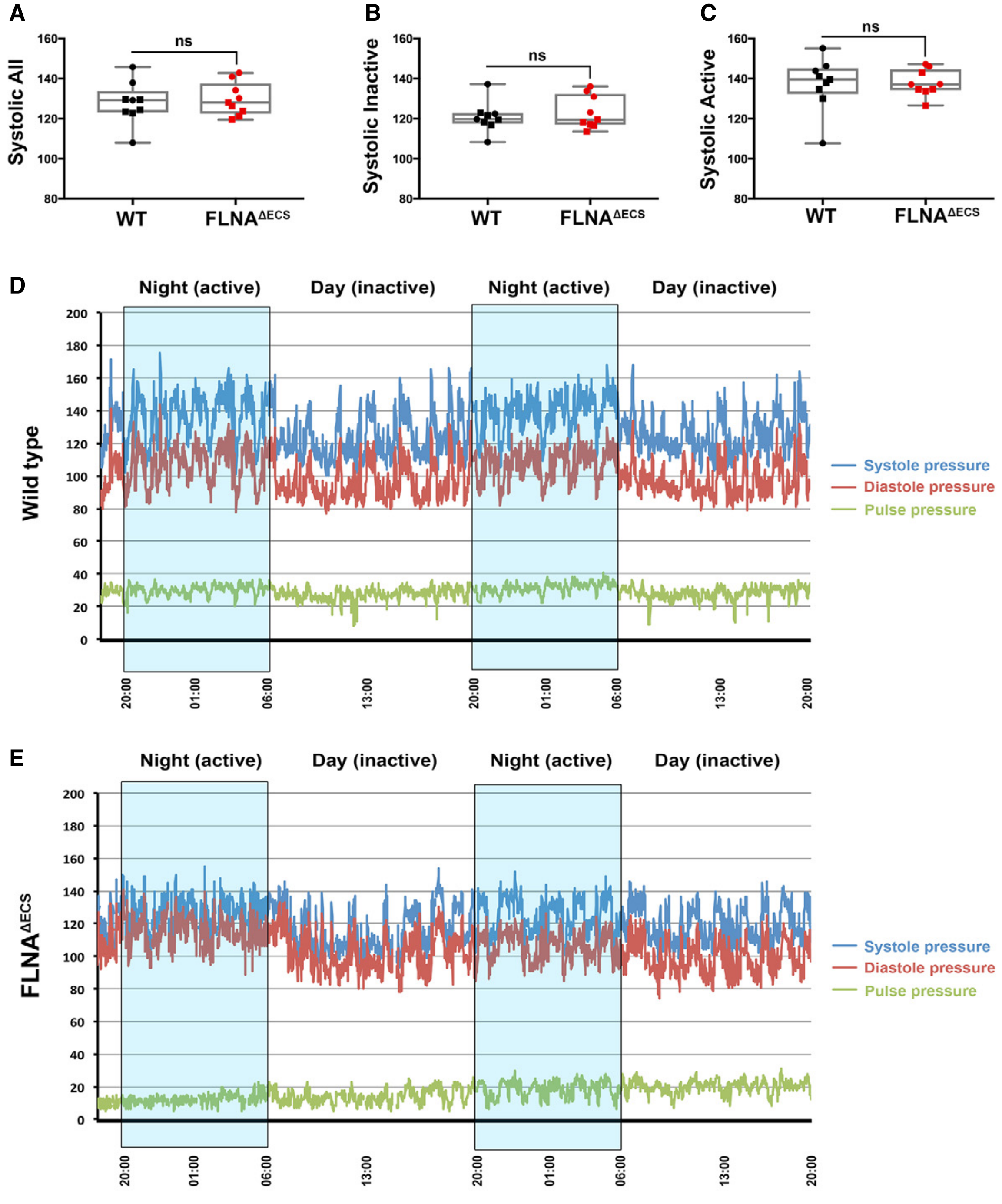

Figure EV5. Long-term telemetric blood pressure measurements in FLNA$^{\Delta ECS}$ and wild-type mice.

A–C Scatter plots showing the systolic blood pressure during a total of 72 h (A), systolic blood pressure during inactive phase (B), and systolic blood pressure during active phase (C). Nine mice were analyzed for each genotype, and the average value is plotted for each mouse. Data are shown as mean ± SEM. Boxes represent the 25th and the 75th percentile with median represented by the black line in the box. The whiskers depict the minimum and the maximum value. *P*-value < 0.05 (Student's *t*-test) was considered significant.

D, E Representative wt (D) and FLNA$^{\Delta ECS}$ (E) mouse showing systolic (blue line) and diastolic (red line) blood pressure for 48 h. The pulse pressure (difference between systolic and diastolic blood pressure) is also indicated (green line). Nighttime (high activity) windows are indicated by blue boxes. In particular, during the resting daylight phases, the FLNA$^{\Delta ECS}$ mice fail to lower their diastolic blood pressure.

Source data are available online for this figure.
