# Supplementary figures and images for: RNA editing of Filamin A pre‐mRNA regulates vascular contraction and diastolic blood pressure
Source: EMBO J. 2018 Aug 7;37(19):e94813. doi: 10.15252/embj.201694813 (PMC6166124; doi:10.15252/embj.201694813)

**Fig 2D**

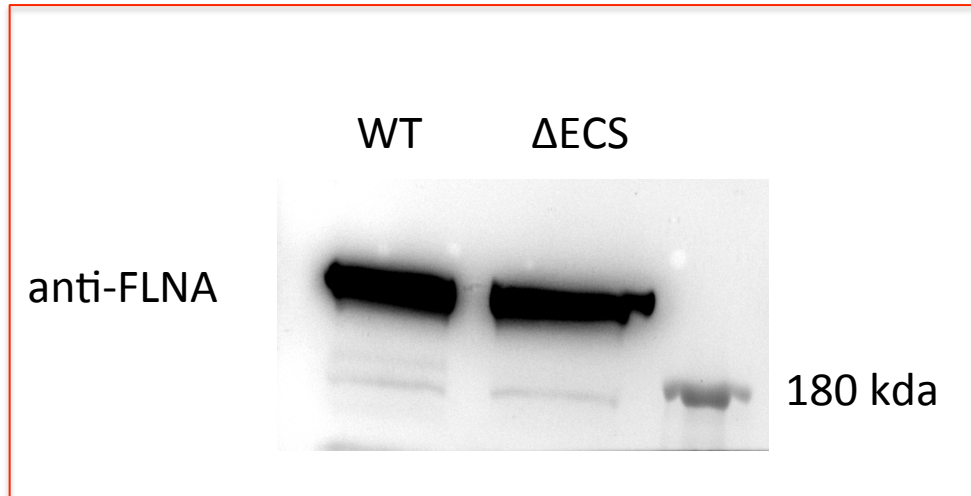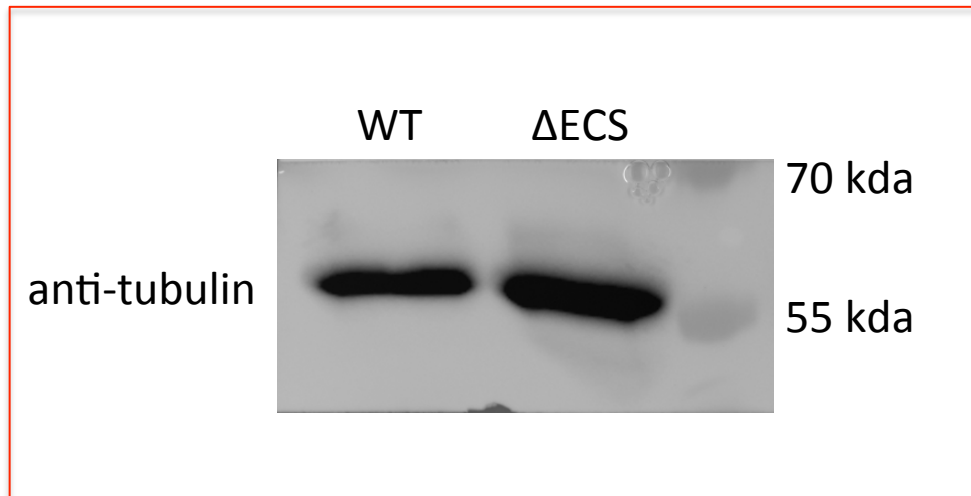

Supplement: Supplementary file 9 — Source Data for Figure 2 [file EMBJ-37-e94813-s007.zip › embj201694813-sup-0006-SDataFig2D.pdf]

**Fig 4A**

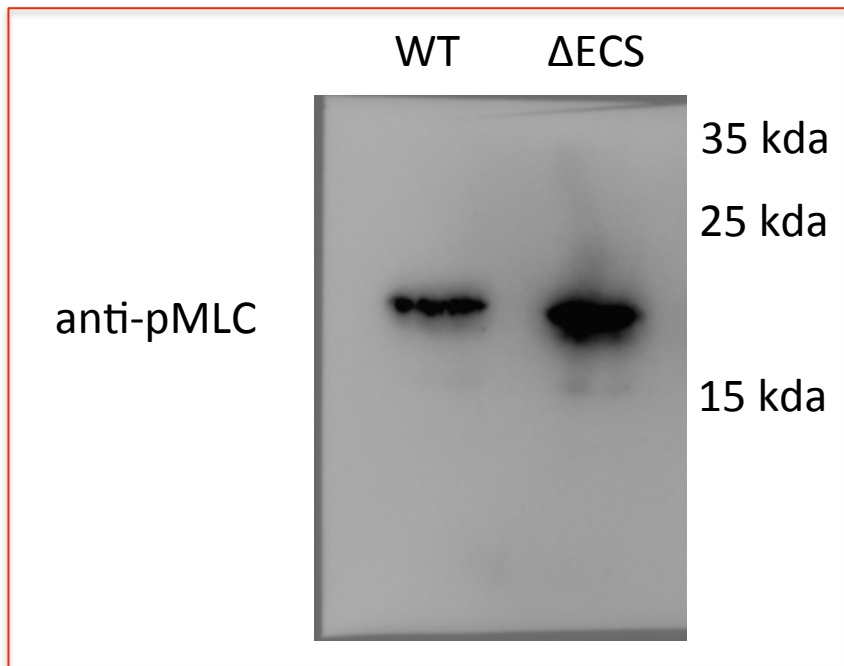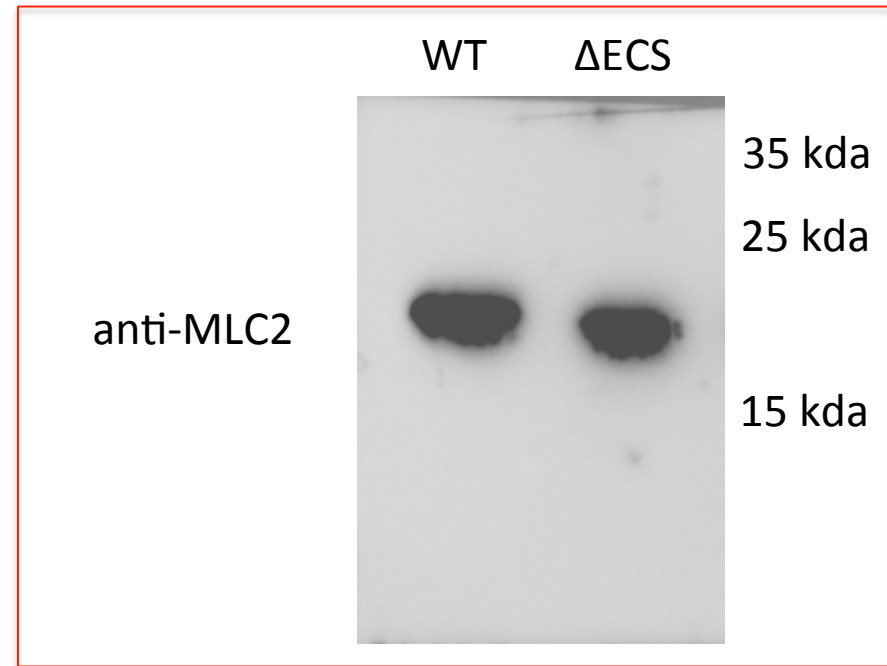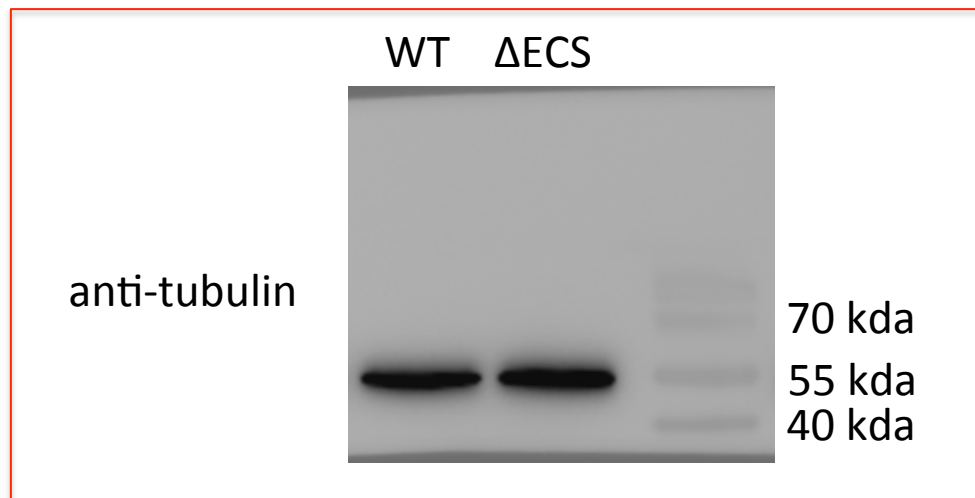

Fig 4B

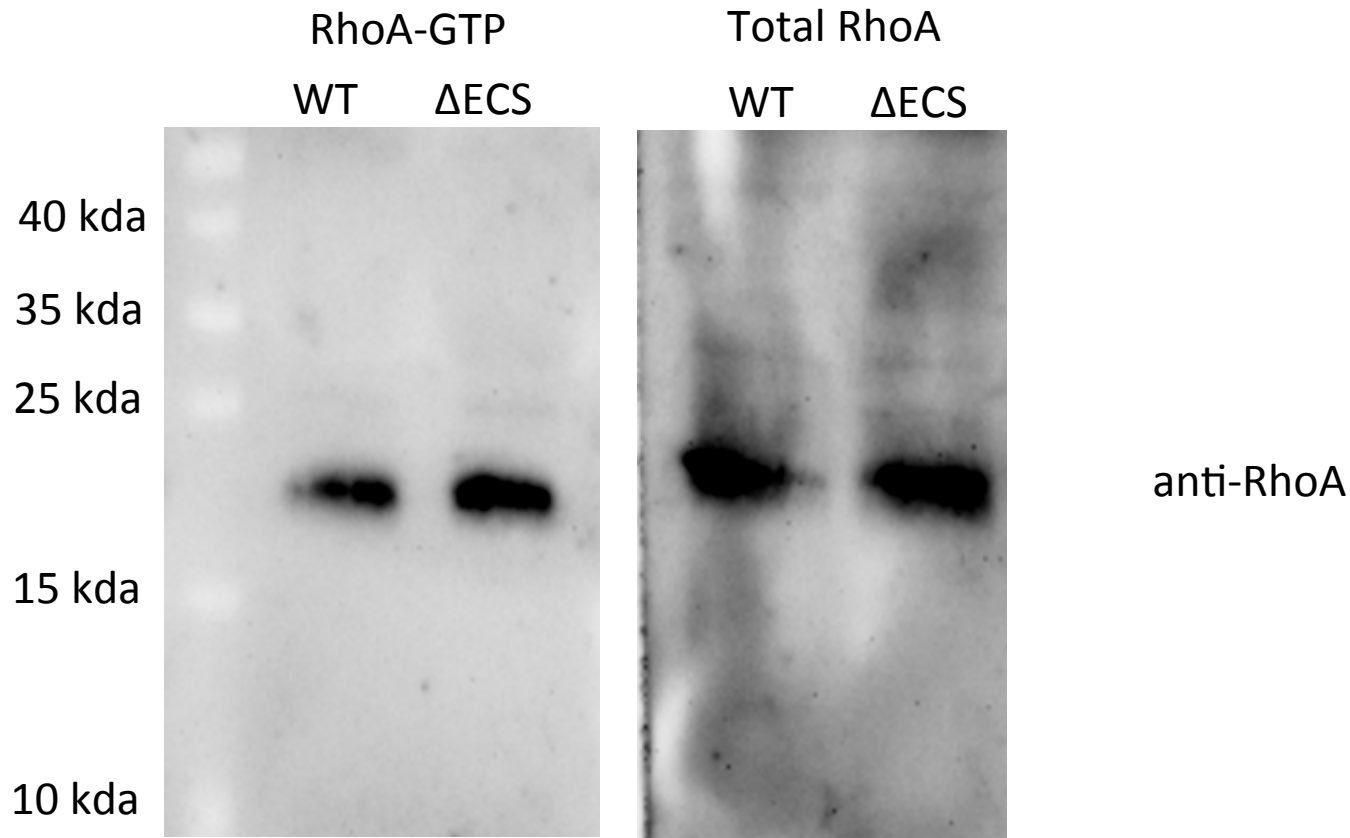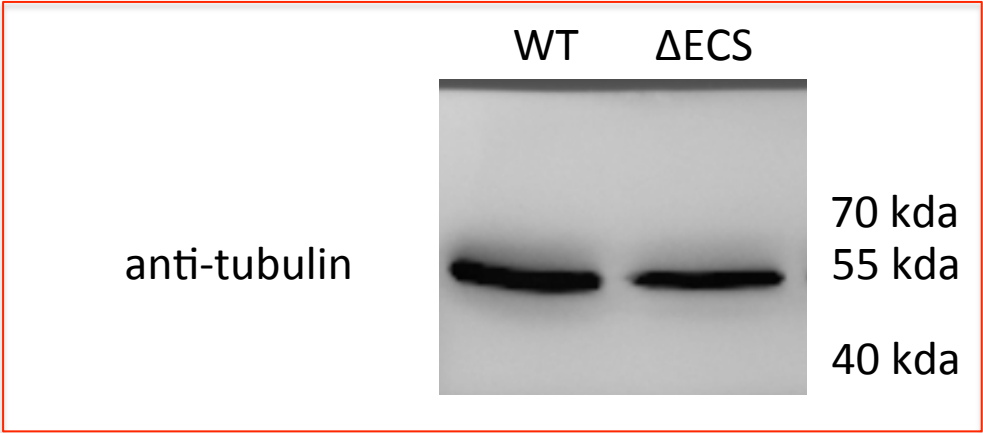

**Fig 4C**

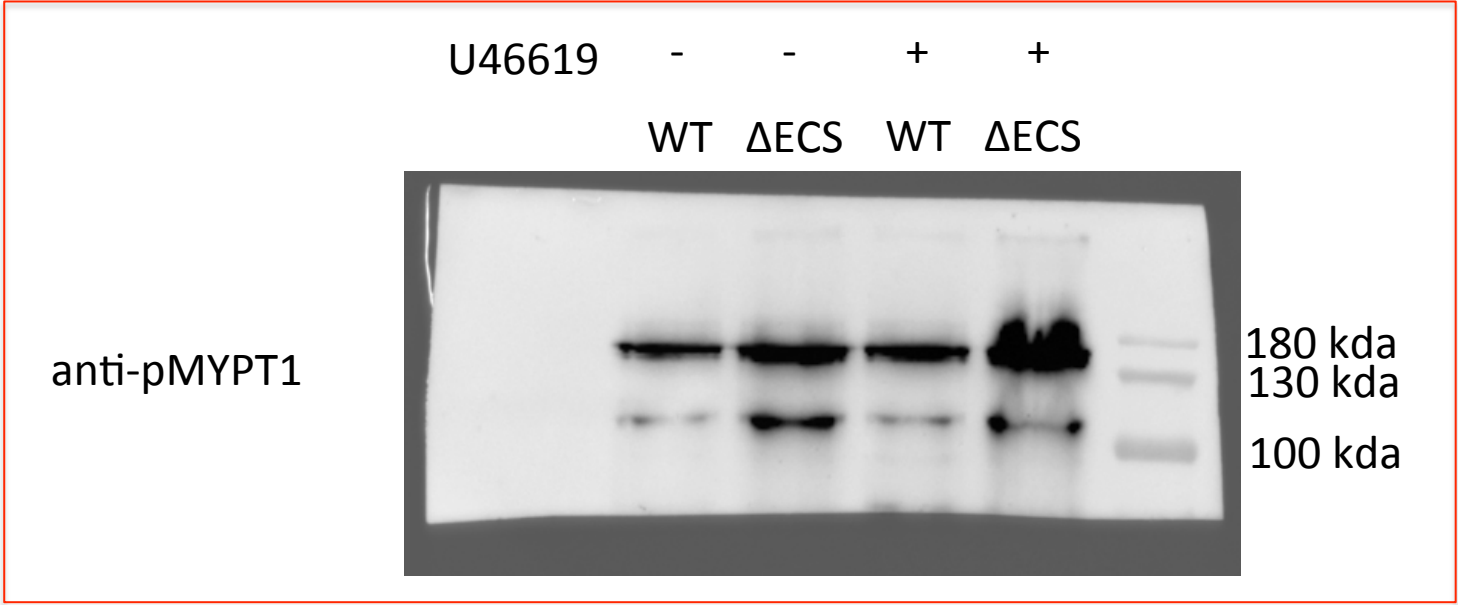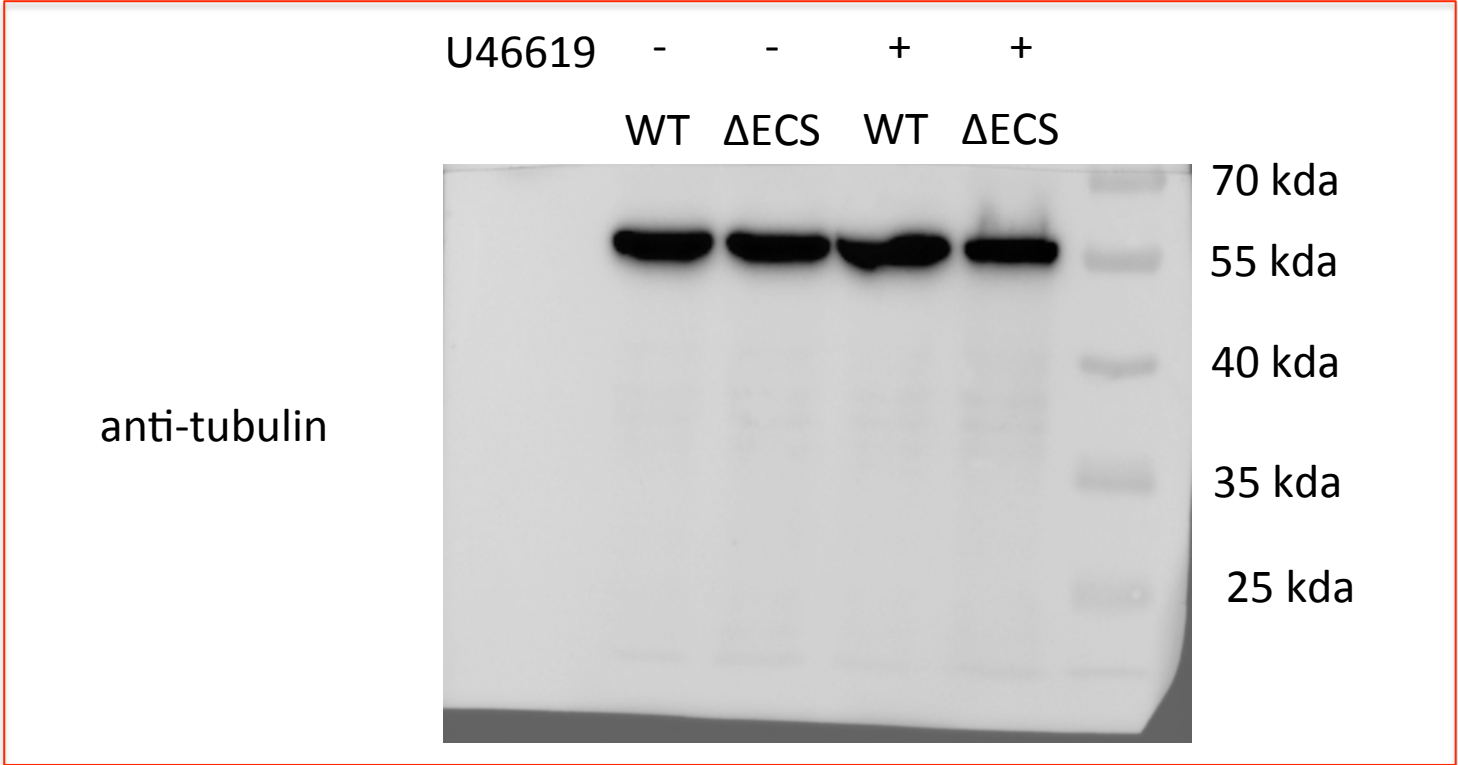

**Fig 4D**

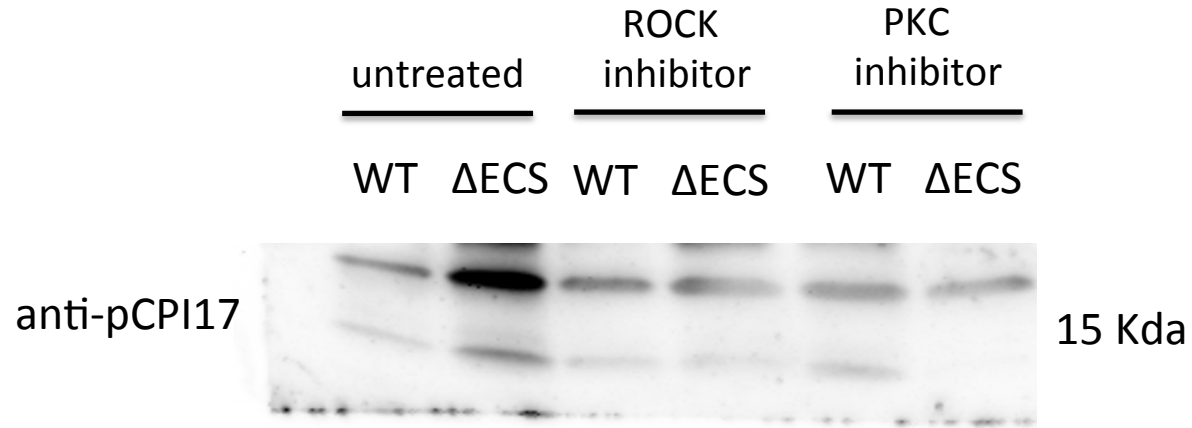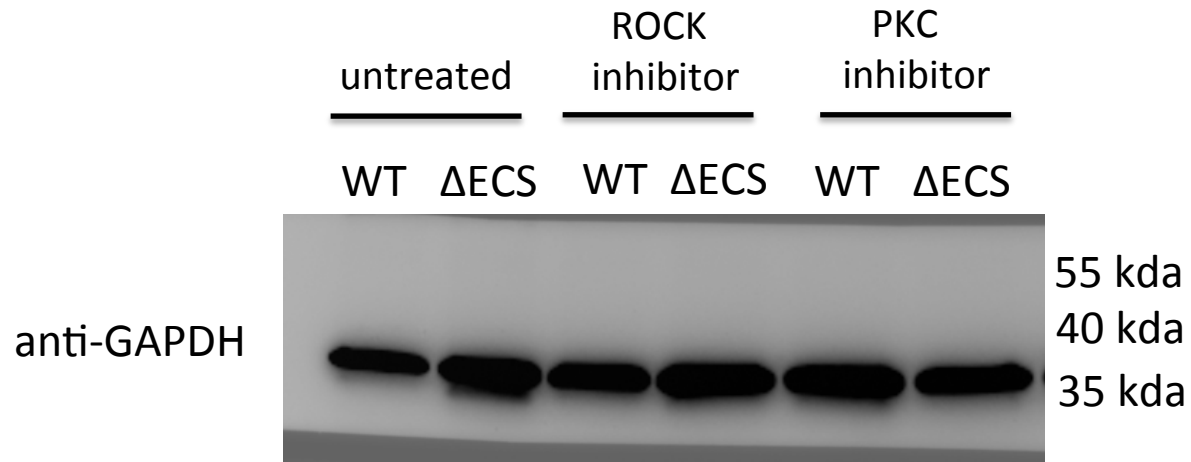

Supplement: Supplementary file 11 — Source Data for Figure 4 [file EMBJ-37-e94813-s009.zip › embj201694813-sup-0009-SDataFig4.pdf]
